# Reduced anticoagulation targets in extracorporeal life support (RATE): study protocol for a randomized controlled trial

**DOI:** 10.1186/s13063-022-06367-w

**Published:** 2022-05-16

**Authors:** Olivier van Minnen, Annemieke Oude Lansink-Hartgring, Bas van den Boogaard, Judith van den Brule, Pierre Bulpa, Jeroen J. H. Bunge, Thijs S. R. Delnoij, Carlos V. Elzo Kraemer, Marijn Kuijpers, Bernard Lambermont, Jacinta J. Maas, Jesse de Metz, Isabelle Michaux, Ineke van de Pol, Marcel van de Poll, S. Jorinde Raasveld, Matthias Raes, Dinis dos Reis Miranda, Erik Scholten, Olivier Simonet, Fabio S. Taccone, Frederic Vallot, Alexander P. J. Vlaar, Walter M. van den Bergh

**Affiliations:** 1 Department of Critical Care, University Medical Center Groningen, Room R3.904, PO BOX 30001, 9700 RB Groningen, The Netherlands; 2 Department of Intensive Care, OLVG, Amsterdam, The Netherlands; 3 Department of Intensive Care Medicine, Radboud University Medical Center, Nijmegen, The Netherlands; 4 Department of Intensive Care, CHU UCL Namur site Mont-Godinne, Yvoir, Belgium; 5 Department of Intensive Care Medicine, Erasmus Medical Center, Rotterdam, The Netherlands; 6 Department of Intensive Care Medicine, Maastricht University Medical Center, Maastricht, The Netherlands; 7 Department of Intensive Care Medicine, Leiden University Medical Center, Leiden, The Netherlands; 8 Department of Intensive Care Medicine, Isala Clinics, Zwolle, The Netherlands; 9 Department of Intensive Care, CHU Sart Tilman, Liege, Belgium; 10 Department of Intensive Care Medicine, St. Antonius Hospital, Nieuwegein, The Netherlands; 11 Department of Intensive Care Medicine, Amsterdam University Medical Center, Location AMC, Amsterdam, The Netherlands; 12 Department of Intensive Care, University Hospital Brussels, Brussels, Belgium; 13 Department of Intensive Care, Centre Hospitalier de Wallonie Picarde (CHwapi), Tournai, Belgium; 14 Department of Intensive Care, Hôpital Erasme Bruxelles, Brussels, Belgium

**Keywords:** ECMO, Anticoagulation, Complications

## Abstract

**Background:**

Although life-saving in selected patients, ECMO treatment still has high mortality which for a large part is due to treatment-related complications. A feared complication is ischemic stroke for which heparin is routinely administered for which the dosage is usually guided by activated partial thromboplastin time (aPTT).

However, there is no relation between aPTT and the rare occurrence of ischemic stroke (1.2%), but there is a relation with the much more frequent occurrence of bleeding complications (55%) and blood transfusion. Both are strongly related to outcome.

**Methods:**

We will conduct a three-arm non-inferiority randomized controlled trial, in adult patients treated with ECMO. Participants will be randomized between heparin administration with a target of 2–2.5 times baseline aPTT, 1.5–2 times baseline aPTT, or low molecular weight heparin guided by weight and renal function. Apart from anticoagulation targets, treatment will be according to standard care. The primary outcome parameter is a combined endpoint consisting of major bleeding including hemorrhagic stroke, severe thromboembolic complications including ischemic stroke, and mortality at 6 months.

**Discussion:**

We hypothesize that with lower anticoagulation targets or anticoagulation with LMWH during ECMO therapy, patients will have fewer hemorrhagic complications without an increase in thromboembolic complication or a negative effect on their outcome. If our hypothesis is confirmed, this study could lead to a change in anticoagulation protocols and a better outcome for patients treated with ECMO.

**Trial registration:**

ClinicalTrials.gov NCT04536272. Registered on 2 September 2020. Netherlands Trial Register NL7969

## Administrative information

Note: the numbers in curly brackets in this protocol refer to SPIRIT checklist item numbers. The order of the items has been modified to group similar items (see http://www.equator-network.org/reporting-guidelines/spirit-2013-statement-defining-standard-protocol-items-for-clinical-trials/).

**Table:** 

Title {1}	Reduced anticoagulation targets in extracorporeal membrane oxygenation (RATE) - a non-inferiority RCT
Trial registration {2a and 2b}.	UMCG research register number 201900659 Clinicaltrials.gov register number NCT04536272 Netherlands trial register number NL7969
Protocol version {3}	1.2
Funding {4}	The Netherlands Organization for Health Research and Development (ZorgOnderzoek Nederland en het gebied Medische Wetenschappen (ZonMw)) project number: 848018014
Author details {5a}	Olivier van Minnen^1^, Annemieke Oude Lansink-Hartgring^1^, Bas van den Boogaard^3^, Judith van den Brule^8^, Pierre Bulpa^14^, Jeroen J. H. Bunge^4^, Thijs S. R. Delnoij^6^, Carlos V. Elzo Kraemer^5^, Marijn Kuijpers^7^, Bernard Lambermont^11^, Jacinta J. Maas^5^, Jesse de Metz^3^, Isabelle Michaux^14^, Ineke van de Pol^9^, Marcel van de Poll^6^, S. Jorinde Raasveld^2^, Matthias Raes^13^, Dinis dos Reis Miranda^4^, Erik Scholten^9^, Olivier Simonet^12^, Fabio S. Taccone^10^, Frederic Vallot^12^, Alexander P. J. Vlaar^2^, Walter M. van den Bergh^1^ ^1^Department of Critical Care, University Medical Center Groningen, Groningen, The Netherlands ^2^Department of Intensive Care Medicine, Amsterdam University Medical Center, Location AMC, Amsterdam, The Netherlands ^3^Department of Intensive Care, OLVG, Amsterdam, The Netherlands ^4^Department of Intensive Care Medicine, Erasmus Medical Center, Rotterdam, The Netherlands ^5^Department of Intensive Care Medicine, Leiden University Medical Center, Leiden, The Netherlands ^6^Department of Intensive Care Medicine, Maastricht University Medical Center, Maastricht, The Netherlands ^7^Department of Intensive Care Medicine, Isala Clinics, Zwolle, The Netherlands ^8^Department of Intensive Care Medicine, Radboud University Medical Center, Nijmegen, The Netherlands ^9^Department of Intensive Care Medicine, St. Antonius Hospital, Nieuwegein, The Netherlands ^10^Department of Intensive Care, Hôpital Erasme Bruxelles, Belgium ^11^Department of Intensive Care, CHU Sart Tilman, Liege, Belgium ^12^Department of Intensive Care, Centre Hospitalier de Wallonie Picarde (CHwapi), Tournai, Belgium ^13^Department of Intensive Care, University Hospital Brussels, Bruxelles, Belgium ^14^Department of Intensive Care, CHU UCL Namur site Mont-Godinne, Yvoir, Belgium
Name and contact information for the trial sponsor {5b}	UMCG government Hanzeplein 1, 9713GZ Groningen, the Netherlands
Role of sponsor {5c}	The UMCG government will have no role in the design of the study and collection, analysis, and interpretation of data and writing the manuscript.

## Introduction

### Background and rationale {6a}

Extracorporeal life support (ECLS) through extracorporeal membrane oxygenation (ECMO) utilization can support the heart and lung for an extended period, up to months, and is deployed in the intensive care unit (ICU) [1]. ECMO seems an efficient therapy in terms of survival benefit, but mortality is still high [2]. This is in part due to the condition that necessitates ECMO, but there is also significant treatment-related mortality. Exposure of blood to the nonbiologic surfaces of an extracorporeal circuit initiates a complex inflammatory response involving both the coagulation and the inflammatory response pathway. A feared complication is a thromboembolic stroke due to clotting related to the ECMO system. To prevent this, and to preserve ECMO circuit patency, patients are treated with systemic anticoagulation, usually with unfractionated heparin (UFH) of which the dosage is guided by activated partial thromboplastin time (aPTT) target of 2.0–2.5 times baseline (approximately 60–75 s). This target is adapted from other diseases or indications for therapeutic anticoagulation and not validated. In recent years ECMO equipment has been improved, e.g., heparin-coated cannulas, but anticoagulation targets remained unchanged [2]. More importantly, there seems to be no relationship between the level of anticoagulation and the occurrence of a thromboembolic stroke. In contrast, there is however a strong relationship between the level of anticoagulation and the occurrence of bleeding complications as well as the need for a blood transfusion which is directly related to poor outcome. Moreover, fatal hemorrhagic stroke is far more frequent than fatal thromboembolic stroke [1, 3]. Taken together, one might postulate that intensive heparin treatment, in this case, might lead to more problems than benefits. However, there is a paucity of studies evaluating different anticoagulation strategies in patients supported with ECMO and no randomized trials are comparing one strategy to another [4–7]. A comprehensive guideline for the use and monitoring of anticoagulation during ECMO therapy may be found on the Extracorporeal Life Support Organization (ELSO) website [8]. This guideline stops short of any mandate, given the lack of evidence in favor of most of the practices reviewed. Rigorous evaluations of anticoagulation use in ECMO patients are therefore urgently needed.

## Objectives {7}

Our primary research question is if anticoagulation with UFH with reduced anticoagulation targets or anticoagulation with low molecular weight heparin (LMWH) leads to a reduction in the occurrence of major bleeding without an increase in thromboembolic complications or a negative effect on outcome compared to the standard practice of high anticoagulation targets with UFH. We expect fewer complications and improvement of survival after ECMO therapy for both interventions.

## Trial design {8}

We will perform a multi-center phase 3, three-armed, randomized, non-inferiority open-label study in patients receiving veno-venous (VV) and veno-arterial (VA) ECMO therapy. Allocation is in a 1:1:1 ratio.

## Methods: participants, interventions, and outcomes

### Study setting {9}

This study will be conducted on the intensive care units of over ten Dutch and Belgian ECMO referral centers. A complete list of all participating hospitals can be found on clinicaltrials.gov.

### Eligibility criteria {10}

All adults receiving ECMO treatment at the ICU of one of the participating centers during the study period are eligible for inclusion.

The exclusion criteria are as follows: (1) patients in whom the ECMO is only used to bridge a procedure like a high-risk percutaneous coronary intervention or during surgery; (2) no (deferred) informed consent; (3) vital indication for robust anticoagulation (e.g., mechanic mitral valve, pulmonary embolism, a clot in the cardiac ventricle); and (4) a history of heparin-induced thrombocytopenia (HIT).

### Who will take informed consent? {26a}

The study intervention regards an emergency intervention that has to be applied without delay and fulfills the ethical requirement of clinical equipoise. The study participant can benefit from the intervention, but up to now, there is a state of honest, professional disagreement in the community of expert practitioners as to the preferred treatment. Furthermore, the eligible patients have an extremely high risk of death and the legal representatives will therefore be in a disturbed mental state complicating an immediately informed decision. For the present study, the local investigator or research nurse will inform the patient about the study intervention if and when his consciousness recovers. As the patient usually remains unable to communicate for several days to weeks, the legal representative is contacted as soon as possible and asks for deferred proxy consent for use of the study data. The rationale for the deferred consent procedure is the clinical equipoise of the interventions, the emergency of the intervention, and the possible benefit for the patient with a positive benefit-risk ratio. If the patient has died before deferred consent could reasonably be obtained, the study data will still be used.

### Additional consent provisions for collection and use of participant data and biological specimens {26b}

This trial does not involve collection of biological specimens.

### Interventions

#### Explanation for the choice of comparators {6b}

We hypothesize that lower anticoagulation targets will result in fewer complications and a better outcome for patients receiving ECMO therapy. We have added the LMWH group because this kind of anticoagulation could provide a more stable level of anticoagulation. Moreover, the safety and potential superiority of subcutaneously administered LMWH compared to intravenous UFH have been described for therapeutic anticoagulation in critically ill patients [9]. LMWH is therefore the method of choice for anticoagulation in the ICU and has been demonstrated to be safe in other extracorporeal circuits such as renal replacement therapy and left ventricular assist device (LVAD) [10]. A disadvantage of LMWH may be the monitoring of the level of anticoagulation with anti-fXa levels instead of aPTT, which may be less reliable.

#### Intervention description {11a}

Eligible patients will be randomized in a 1:1:1 ratio between three anticoagulation strategy’s during ECMO therapy: (1) UFH with a target of 2–2.5× baseline aPTT (usual care, about 60–75 s), (2) UFH with a target of 1.5–2.0× (45–60 s), and (3) therapeutic dosage LMWH guided by weight and renal function. Adjustment of heparin and dosage of LMWH will be done according to the local anticoagulation protocols of the participating centers. After the start of ECMO timing of anticoagulation administration will be done according to usual care, so maybe postponed in case of postoperative bleeding for up to 24 hours. No additional invasive procedures are performed in the course of this research. Only laboratory or radiological tests that are part of routine medical treatment will be obtained if relevant.

#### Criteria for discontinuing or modifying allocated interventions {11b}

Crossover to another treatment arm than allocated is allowed based on any of the following: (1) meeting one of the components of the primary outcome, (2) an acquired vital indication for high therapeutic anticoagulation, and (3) additional laboratory results that point towards a strong risk for coagulation disorder or hypercoagulation. Crossover to another treatment than allocated is in principle temporary. The timing of reversion is left to the consideration of the treating physician. The crossover will be mentioned in the case report form (CRF) and the primary and secondary outcome parameters will be measured and mentioned in the CRF after crossover as well.

#### Strategies to improve adherence to interventions {11c}

On-site kick-off meetings are planned before the start of recruitment with the local investigators, research nurses, and treating physicians to explain our hypothesis and the need for this study and will be repeated yearly. By this, we believe the adherence to the study protocol and recruitment overall will be optimal. The monitoring plan will be described at point 23.

#### Relevant concomitant care permitted or prohibited during the trial {11d}

Implementing of three anticoagulation strategy’s during ECMO support will not require alteration to concomitant usual care pathways (including use of any medication) and these will continue for all three trial arms.

#### Provisions for post-trial care {30}

There is no anticipated harm and compensation for trial participation.

### Outcomes {12}

The primary outcome parameter of this study is a composite endpoint consisting of (1) severe hemorrhagic complications according to the ELSO definitions: clinically overt bleeding with a transfusion requirement of more than 20 ml/kg red blood cell (RBC) transfusions or >3U RBC in one calendar day. Bleeding that is retroperitoneal or pulmonary or involves the central nervous system or bleeding that requires surgical intervention will also be considered major bleeding; (2) severe thromboembolic complication defined as ischemic stroke, limb ischemia, or acute pump failure; (3) mortality at 6 months. This composite outcome was designed to capture the net clinical effect of reduced anticoagulation targets, e.g., a reduction of major bleeding not counteracted by an increase in thromboembolic complications. Mortality is part of the composite outcome to capture unknown or unmeasured effects of reduced anticoagulation.

Secondary endpoints are all other variables that may be affected by the anticoagulation regime: (1) blood transfusions, (2) quality of life (HR-QoL) at 6 months, (3) exchange of the membrane oxygenator, (4) vessel thrombosis after ECMO removal detected by echography, (5) pulmonary embolism, (6) costs, (7) the individual components of the composite outcome, (8) all thromboembolic complications combined, and (9) all hemorrhagic complications combined.

### Participant timeline {13}

**Fig. 1:**
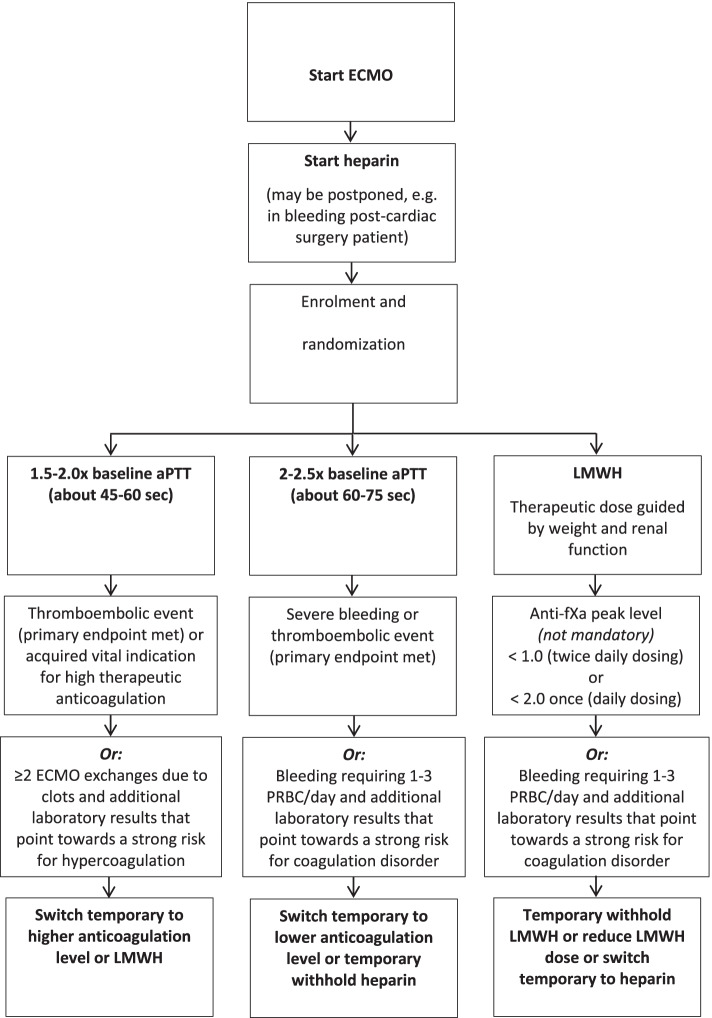
Flowchart. Decision tree randomization and crossover

See the flowchart in Fig. 1.

### Sample size {14}

We expect that with a target of 1.5–2.0× baseline aPTT or with LMWH the primary composite endpoint will be reached in 60% of patients compared to 70% in usual care. To show non-inferiority with a significance level (alpha) of 5%, power of 80%, and a non-inferiority limit (delta) of 7.5%, the corresponding sample size is 91 patients per group. In other words, if there is a true difference in favor of the experimental treatment of 10%, then 91 patients per group are required to be 80% sure that the upper limit of a one-sided 95% confidence interval (or equivalently a 90% two-sided confidence interval) will exclude a difference in favor of the standard group of more than 7.5%, thus 75.25% in the intervention groups compared to 70% in usual care. To compensate for a lower effect and drop-outs, 330 patients will be enrolled. Drop-outs are defined as the withdrawal of informed consent or loss to follow-up.

### Recruitment {15}

All adult patients who receive ECMO treatment during the study period in one of the participating centers can be considered for enrolment; they will be included and randomized by local investigators. As previously mentioned, on-site kick-off meetings are planned before the start of recruitment with the local investigators, research nurses, and treating physicians to explain our hypothesis and the need for this study and will be repeated yearly, to improve recruitment.

## Assignment of interventions: allocation

### Sequence generation {16a}

All patients who receive ECMO treatment during the study period in one of the participating centers can be considered for enrolment in the study. Randomization will be 1:1:1, using variable block size and stratified by ECMO mode and study site.

### Concealment mechanism {16b}

Randomization will be performed if the subject meets all inclusion and exclusion criteria and will be processed centrally using a web-based system that will provide the randomization treatment arm (a target of 2–2.5× baseline aPTT, 1.5–2.0×, or therapeutic LMWH). The online system (ALEA Research®) is constructed and validated for randomization and data management and has an audit trail.

### Implementation {16c}

Contact persons of all participating centers can sign in to the web-based randomization system and randomize their patients. Patients will be automatically allocated to one of the anticoagulation regimes by the system.

## Assignment of interventions: blinding

### Who will be blinded {17a}

In this single-blind open-label study, only the patient will be blinded for the allocation. Outcome assessment and statistical analysis will be done blinded for treatment allocation.

### Procedure for unblinding if needed {17b}

As the design is open-label, that is single-blind, no indications for breaking the randomization code are provided in the protocol. Randomization is communicated with the local principal investigator of each participating hospital who further carries out the necessary arrangements. Based on the aPTT levels achieved concealed allocation can be controlled.

## Data collection and management

### Plans for assessment and collection of outcomes {18a}

Data will be collected and stored as described at point 19. Data will be collected from the patients’ medical files and entered into the eCRF. After 6 months, patients who are still alive will be contacted by phone for health-related quality of life questionnaire (EQ-5D-5L). The EQ-5D-5L questionnaire consists of 5 questions in 5 domains: mobility, self-care, usual activities, pain/discomfort, and anxiety/depression. Each dimension has 5 levels. The answers to this questionnaire result in a 5-digit number that describes the patients’ health state. Another part of the questionnaire is the EQ-VAS which records the patients’ self rated-health on a scale from 0 to 100. The EQ-5D is the most widely used health-related quality of life questionnaire in health economic evaluations [11].

### Plans to promote participant retention and complete follow-up {18b}

As mentioned at 11b, crossover to another treatment arm than allocated is allowed and in principle temporary. The timing of reversion is left to the consideration of the treating physician. The crossover will be mentioned in the CRF, and the primary and secondary outcome parameters will be measured and mentioned in the CRF after crossover as well. In case of no informed consent, the patient will no longer be exposed to the allocated study intervention, but consent will be asked to collect the data according to the study protocol. If no informed consent is given for this, the patient will be withdrawn from the study and replaced. Quality of life assessment will be recorded by means of a structured telephone interview. If the patient does not answer the phone, several attempts will be made to contact again.

### Data management {19}

For each randomized patient, a digital CRF will be formed. Central data management will be performed in REDCap (Research Electronic Data Capture) by technicians and data managers of the trial coordination center. Trial data will be entered in the patient’s CRF by the local investigator or research nurse. REDCap gives multiple tools to promote data quality. The project leader will screen REDCap regularly for missing and incorrect data. When present the project leader will contact the local investigator to adjust or complete the eCRF. The data will be kept for at least 25 years.

### Confidentiality {27}

All randomized patients are identified by a patient independence number in combination with a center number. Trial personnel will not pass names outside the local hospital. On screening forms, digital CRF, or other documents submitted to the coordinating center, patients will not be identified by their names but by their numbers. The subject identification code will be safeguarded by the local investigator.

### Plans for collection, laboratory evaluation, and storage of biological specimens for genetic or molecular analysis in this trial/future use {33}

See above 26b; no biological specimen will be collected.

## Statistical methods

### Statistical methods for primary and secondary outcomes {20a}

#### Primary study parameters

The primary analysis will be a single comparison between the treatment groups of the primary outcome measure after 6 months. This analysis will be performed according to the intention-to-treat principle. To assess the effect of treatment with lower anticoagulation targets or LMWH with standard care, an absolute risk reduction of poor outcome and its corresponding 95% confidence interval will be calculated. The confidence interval of the risk reduction will be compared with the non-inferiority marge of 7.5% of both intervention arms adjusted for center and ECMO mode.

#### Secondary study parameters

For secondary outcome measures, between-group differences will be analyzed using independent samples *t*-tests, chi-square test, or Mann-Whitney tests, where appropriate and adjusted for center and ECMO mode. If necessary, multivariable regression analysis will be used to adjust for imbalances in main prognostic variables between the intervention and control groups.

#### Other study parameters

##### Cost-effectiveness analyses

The difference in costs for use of heparin and aPTT measurements will be neglectable. Cost-effectiveness will be based on reduced costs of blood transfusions and interventions for bleeding as well as improved outcome. All medical cost items expected to be affected by the ECMO therapy will be measured and valued according to the Dutch and Belgium standard guidelines for economic evaluations, e.g., blood transfusion, number of ECMO replacements, surgery, and hospital length of stay. Health gains will be measured in terms of quality-adjusted life-year (QALY) based on EQ-5D-5L-defined utilities. The Budget Impact Analysis (BIA) will be performed from a healthcare perspective to inform decision-makers about the financial consequences of reduced anticoagulant targets in ECMO treatment in Dutch and Belgium healthcare. The model will take changes in the availability and adoption of the reduced anticoagulant targets into account by calculating the financial consequences of five scenarios with a time horizon of 5–10 years.

### Interim analyses {21b}

An unblinded interim analysis will be performed when the first 150 patients have been enrolled in the study. An independent statistician will perform this analysis and the results will be presented to the safety committee. The commission will calculate the power for non-inferiority, conditioned on the difference between treatments concerning outcome rates and on the non-inferiority margin of 7.5%. If the conditional power is 50–79%, the sample size will be re-estimated to maintain a conditional power of 80%.

If the difference between the treatment groups will be significant at an alpha level of 1%, the trial will be stopped because of “proof beyond reasonable doubt” that intervention treatment is non-inferior to standard treatment.

### Methods for additional analyses (e.g., subgroup analyses) {20b}

There will be no per protocol subgroup analysis. We will perform a comparison between the treatment groups adjusted for medical center and ECMO mode.

### Methods in analysis to handle protocol non-adherence and any statistical methods to handle missing data {20c}

Protocol violations will be reported in the investigator site files. The principal investigator will screen the eCRF regularly for missing data and will encourage local investigators to complete the eCRF. Nonadherence to the allocated treatment arm is possible as described at 11b; the subject will be analyzed as in the original treatment arm, according to the intention-to-treat principle.

### Plans to give access to the full protocol, participant-level data, and statistical code {31c}

The protocol of the study is publicly available on the website: https://ecmo-nl.com. The dataset generated during this study is available from the corresponding author upon reasonable request.

## Oversight and monitoring

### Composition of the coordinating center and trial steering committee {5d}

The principal investigator will have overall responsibility for the study and its management. The trial executive committee that consists of the principal investigator, project coordinator, research nurse, and data manager are all affiliated with the University Medical Center Groningen. They will be responsible for the day-to-day running of the trial. The trial steering committee will meet biannually or more often when needed. The study is part of the Dutch ECLS Study Group in which all Dutch ECMO centers are represented.

### Composition of the data monitoring committee, its role and reporting structure {21a}

The study is considered a low-risk study and therefore we have appointed a safety committee instead of a Data Monitoring Committee (DMC) to perform interim analyses for safety, futility, or positive efficacy so that the steering committee can remain blinded for the outcome of the study. All the members have no conflict of interest with the sponsor of the study.

The Safety Committee will:

- Monitor recruitment figures and losses to follow-up
- Monitor evidence for treatment harm
- Monitor overall conduct and data quality, including completeness, encouraging collection of high-quality data
- Make recommendations that the trial continues to recruit participants or whether recruitment should be terminated either for everyone or some treatment groups and/or some participant subgroups
- Perform the pre-planned interim analysis and recommend the continuation of the study accordingly
- Suggest additional data analyses
- Give uncalled-for recommendations based for example on data from recently presented studies
- Give uncalled-for recommendations if the assumptions made for the sample size calculation of the study prove to be incorrect. The assumptions may pertain to patient accrual and the incidence of the primary outcome event.

The executive committee will send a safety report to the safety committee once every 3 months. The advice of the safety committee will be sent to the sponsor of the study and the reviewing Medical Research Ethics Committee (MREC).

### Adverse event reporting and harms {22}

Clinical research involving critically ill patients illustrates several concerns with the existing system for monitoring adverse events.

Morbidity and mortality rates are high among patients in the ICU. In this particular study population, mortality rates exceed 50% [12]. Therefore, whether enrolled in a trial or not, ICU patients are particularly likely to experience clinical events that fall within the definition of a serious adverse event. These events include death, nosocomial infection, and laboratory test results indicating potentially dangerous physiologic abnormalities. Thus, a high proportion of ICU patients may experience a serious adverse event.

We clearly described the SAEs we plan to identify and label these as primary and secondary outcomes. These outcomes will be reported in the CRF and do not have to be reported separately. Periodic reports of SAEs will be reported through an Internet portal for submission, review, registration, and publication of medical research involving human subjects, to the accredited MREC that approved the protocol and the safety committee. Since case fatality in the patient population under study is known to be around 50%, a line listing of patients that have met the primary endpoint including deaths will be performed, with reporting once per 3 months. This reporting will be the responsibility of the study coordinator and the primary investigator. Other SAE’s then the outcome measures can be spontaneously reported in the CRF if serious and possible occur as a consequence of the study and will also be included in the 3 monthly line listing report.

### Frequency and plans for auditing trial conduct {23}

Monitoring will be executed in compliance with The Netherlands Federation of University Medical Centers-guideline (NFU) “Quality Assurance of research involving human subjects 2.0” [13]. Monitoring will be performed by an independent and qualified monitor.

To ensure patient’s rights, wellbeing, and safety and compliance as well as the quality of data, the monitor will visit the sites regularly. For this study, the risk classification is considered low (based on the NFU guideline), which implies monitoring of at least 1 visit per site per year. The frequency of the visits depends on the actual patient inclusion rate and the observed events and deviations on a site.

The monitor will verify the following items: patient flow (inclusion and dropout rate); informed consent forms (presence, dates, signatures); Trial Master File and Investigator Site File (presence of all essential documents); in- and exclusion criteria; primary endpoint; SAEs (missed events, reporting procedures); and study treatment (patient instructions, administration, accountability). Source data verification will be performed for a selection of patients on a pre-selected set of data (focused on endpoints and safety). Source documents are defined as the patient’s hospital medical records, clinician notes, laboratory printouts, digital and hard copies of imaging, memos, electronic data, etc.

The monitor will verify the compliance to study procedures, standard operating procedures, and other instructions. The presence of certificates, standard operating procedures, and instructions related to devices, facilities, laboratories, pharmacy, and other departments involved will be checked.

Findings from the monitoring visits will be reported by the monitor to the sponsor-investigator through a monitoring visit report. It is the responsibility of the sponsor-investigator to follow up on findings, deviations, queries, or other issues where required.

### Plans for communicating important protocol amendments to relevant parties (e.g., trial participants, ethical committees) {25}

All substantial amendments will be notified to the MREC and the competent authority. Non-substantial amendments will not be notified to the MREC and competent authority but will be recorded and filled by the sponsor. All amendments will be notified to the local investigators by e-mail and through the study website.

### Dissemination plans {31a}

If our study indeed shows that reduced anticoagulation targets improve outcomes, the next steps will be to disseminate the obtained insights among health care professionals, patients, and policy makers and to implement reduced anticoagulation targets in clinical practice.

Successful implementation starts with involving the various stakeholders from the start. In our study, it is especially relevant to involve both referrers, other professionals (health technology assessment and implementation experts), and patients, and therefore, we included them in our project group. The project group members have a broad network in the Netherlands, are members of the Dutch Society of Intensive Care (NVIC) and represented in the commission ECLS of the NVIC and European Society of Intensive Care Medicine (ESICM), have written the current Dutch guideline for ECLS treatment, and are experts in the field of ECLS therapy. The project group members are well-positioned to guarantee dissemination of the insights obtained among their colleagues, by presenting the results at national and international podia and by writing reports and papers.

## Discussion

This three-armed non-inferiority study will be the first randomized controlled trial investigating the effect of lower anticoagulation targets or anticoagulation with LMWH during ECMO on outcome and complications.

Several coagulation assays can be used to monitor and titrate UFH. In an attempt to reduce high complication rates, many centers have incorporated additional coagulation assays, including aPTT, heparin anti-fXa level, AT activity, and TEG, in their routing monitoring [4, 14–22]. In most international centers including all Dutch centers, patients are preferably treated with systemic anticoagulation using heparin with an aPTT target of 2.0–2.5 times baseline. This target adapted from other indications for therapeutic anticoagulation but has never been validated for use during ECMO. More importantly, there seems to be no relationship between the level of anticoagulation and the occurrence of thromboembolic stroke. However, there is a strong relationship between the level of anticoagulation and the occurrence of hemorrhagic complications [1, 3]. Taken together, intensive UFH therapy seems to do more harm than good. The safety and potential superiority of subcutaneously administered LMWH over UFH has been described in critically ill patients and leads to more balanced anticoagulation [10, 23]. Several small observational studies showed no negative effect on the outcome or increase in complications when reduced anticoagulation during ECMO where used.

Our primary research question is if anticoagulation with UFH with reduced anticoagulation targets or anticoagulation with LMWH leads to a reduction in the occurrence of major bleeding without an increase in thromboembolic complications or a negative effect on outcome compared to the standard practice of high anticoagulation targets with UFH. We expect fewer complications with subsequent medical costs savings and improvement of survival and quality of life 6 months after initiation of ECMO therapy, resulting in fewer costs per QALY for both interventions.

The strengths of this study are the multi-center design of the study, the formulated protocol, the lack of extra interventions besides standard care which will improve protocol adherence, the inclusion of both VV and VA ECMO, and the use of three anticoagulation strategies.

Some limitations should be noted for this study. First, it is unclear whether the level of anticoagulation by UFH can best be monitored with aPTT. However, this method is currently part of the monitoring protocol of all participating hospitals.

Another limitation could be that the level of anticoagulation in the control group may not be very different compared with the lower aPTT target group, just above or below 60 seconds. However, we aim to prove non-inferiority, not superiority, which would have needed a more pronounced difference in aPTT or a much larger sample size.

Titration of the anticoagulation level by dose adjustments of UFH and LMWH will be done according to the local protocol of the participating center. The type of LMWH used also depends on which is used according to the local protocol in the participating center. Because of this, the study can be carried out well and easily in different centers. However, this could potentially influence the outcome of our analysis if one local anticoagulation protocol would be inferior to another. Therefore, we will adjust for center in the statistical analysis to correct potential bias.

This study is the first major step towards more personalized medicine for patients supported by ECMO and creates opportunities for a precision medicine approach. Over the years, all patients supported with ECMO were treated with the same anticoagulation targets. This one-size-fits-all approach may have prevented thromboembolic complications in some patients, but most likely has also resulted in bleeding complications in many others. In recent years with the introduction of improved membrane oxygenators and heparin-coated cannulas, our feeling is that the balance has turned to more harm than benefit. If our study indeed shows that lower anticoagulation targets reduce bleeding complications and improve outcome this would be a huge step towards better care for these patients. Further finetuning of anticoagulation targets will most likely not be established by randomized controlled trials, but with cohort studies in which data obtained in the proposed study will pave the way for researchers and, ultimately, clinicians to be able to target anticoagulation strategies more appropriately and effectively.

## Trial status

The RATE study is currently recruiting in 8 hospitals in the Netherland. The recruitment began in September 2020 and is estimated to be completed in April 2023. To date, 111 participants have been recruited. During the COVID-19 pandemic, fewer participants than expected have been recruited due to several reasons including reduced staffing to perform research. The planned end date is already adapted to the new prognosis and prolonged with permission of the sponsor and funder to recruit the planned 330 patients.

## Abbreviations

AE: Adverse event
aPTT: Activated partial thromboplastin time
BIA: Budget Impact Analysis
CRF: Case report form
DMC: Data monitoring committee
ECLS: Extracorporeal life support
ECMO: Extracorporeal membrane oxygenation
ELSO: Extracorporeal Life Support Organization
HIT: Heparin-induced thrombocytopenia
HR-QoL: Health-related quality of life
ICU: Intensive care unit
LMWH: Low molecular weight heparin
LVAD: Left ventricular assist device
MREC: Medical Research Ethics Committee
NFU: The Netherlands Federation of University Medical Centers
RBC: Red blood cell
QALY: Quality-adjusted life year
SAE: Serious adverse event
UFH: Unfractionated heparin
ZonMw: The Netherlands Organization for Health Research and Development (*ZorgOnderzoek Nederland en het gebied Medische Wetenschappen*)
